# Supplementary material for: Long-Term Outcomes after Stroke in Patients with Atrial Fibrillation: A Single Center Study
Source: Int J Environ Res Public Health. 2023 Feb 16;20(4):3491. doi: 10.3390/ijerph20043491 (PMC9967874; doi:10.3390/ijerph20043491)
Supplement: Supplementary file 1 [file ijerph-20-03491-s001.zip › ijerph-2210648-supplementary.pdf]

Table S1. Multiple regression of outcomes at 1 year, 3 years and 5 years after stroke with all variables.

	Morality						Recurrence					
	Within 1 year		Within 3 years		Within 5 years		Within 1 year		Within 3 years		Within 5 years	
Predictors	*p*	OR	*p*	OR	*p*	OR	*p*	OR	*p*	OR	*p*	OR
Age <65years	0.030		0.026		0.021		0.029		0.026		0.021	
65-74years	0.069	2.02 (0.95-4.33)	0.070	2.02 (0.94-4.33)	0.067	2.04 (0.95-4.36)	0.074	2.00 (0.93-4.27)	0.076	1.99 (0.93-4.27)	0.070	2.02 (0.94-4.31)
> 74years	0.008	2.61 (1.28-5.33)	0.007	2.67 (1.31-5.44)	0.006	2.73 (1.34-5.57)	0.008	2.61 (1.29-5.31)	0.007	2.66 (1.31-5.40)	0.005	2.72 (1.34-5.51)
Atrial fibrillation	0.247	1.59 (0.72-3.51)	0.683	0.82 (0.31-2.15)	0.207	0.36 (0.07-1.76)	0.157	1.85 (0.79-4.33)	0.660	1.36 (0.35-5.34)	0.075	0.19 (0.03-1.18)
Gender	0.503	1.20 (0.71-2.03)	0.504	1.20 (0.71-2.03)	0.465	1.22 (0.72-2.06)	0.447	1.23 (0.72-2.07)	0.452	1.22 (0.72-2.07)	0.450	1.22 (0.72-2.07)
Heart failure	0.005	2.75 (1.35-5.58)	0.005	2.75 (1.36-5.60)	0.004	2.80 (1.38-5.69)	0.005	2.78 (1.37-5.65)	0.005	2.78 (1.36-5.66)	0.005	2.79 (1.37-5.66)
Hypertension	0.468	1.27 (0.66-2.44)	0.483	1.26 (0.66-2.42)	0.491	1.26 (0.66-2.41)	0.467	1.27 (0.66-2.44)	0.473	1.27 (0.66-2.43)	0.492	1.26 (0.66-2.41)
Peripheral arterial disease	0.606	1.19 (0.61-2.33)	0.594	1.20 (0.61-2.34)	0.621	1.18 (0.61-2.32)	0.622	1.18 (0.60-2.32)	0.622	1.18 (0.60-2.32)	0.634	1.18 (0.60-2.30)
Coronary artery disease	0.885	1.04 (0.61-1.78)	0.883	1.04 (0.61-1.78)	0.860	1.05 (0.61-1.79)	0.898	1.04 (0.61-1.77)	0.899	1.04 (0.61-1.77)	0.882	1.04 (0.61-1.78)
Smoking	0.021	0.30 (0.11-0.83)	0.019	0.29 (0.11-0.82)	0.020	0.29 (0.11-0.83)	0.020	0.29 (0.11-0.82)	0.022	0.30 (0.11-0.84)	0.022	0.30 (0.11-0.84)
Alcoholism	0.999	1.00 (0.28-3.57)	0.993	0.99 (0.28-3.56)	0.993	0.99 (0.28-3.56)	0.953	0.96 (0.27-3.49)	0.961	0.97 (0.27-3.53)	0.973	0.98 (0.27-3.52)
Antithrombotics treatment	0.422	0.80 (0.46-1.39)	0.434	0.80 (0.46-1.39)	0.427	0.80 (0.46-1.39)	0.530	0.84 (0.48-1.46)	0.525	0.83 (0.48-1.46)	0.460	0.81 (0.47-1.41)
NIHSS at admission < 7	0.299		0.292		0.263		0.257		0.274		0.266	
8-15	0.219	0.79 (0.40-1.59)	0.533	0.80 (0.40-1.61)	0.557	0.81 (0.41-1.63)	0.564	0.82 (0.41-1.63)	0.580	0.82 (0.41-1.64)	0.569	0.82 (0.41-1.63)
> 16	0.120	0.41 (0.14-1.26)	0.117	0.41 (0.13-1.25)	0.104	0.40 (0.13-1.21)	0.101	0.39 (0.13-1.20)	0.110	0.40 (0.13-1.23)	0.106	0.40 (0.13-1.21)
NIHSS at discharge < 7	0.133		0.123		0.101		0.117		0.116		0.107	
8-15	0.671	0.84 (0.38-1.86)	0.658	0.83 (0.37-1.86)	0.692	0.85 (0.38-1.90)	0.744	0.88 (0.39-1.95)	0.718	0.86 (0.39-1.92)	0.704	0.86 (0.38-1.91)
> 16	0.080	5.36 (0.82-35.09)	0.077	5.40 (0.83-35.05)	0.060	5.92 (0.93-37.81)	0.063	5.90 (0.91-38.26)	0.065	5.82 (0.90-37.72)	0.061	5.85 (0.92-37.30)

Abbreviations: NIHSS, National Institutes of Health Stroke Scale; OR, odds ratio
